# Understanding the Relationship Between Weight Discrimination and Cardiometabolic Risk Factors in Diabetes: Findings from the English Longitudinal Study of Ageing (ELSA)

**DOI:** 10.1007/s12529-025-10421-7

**Published:** 2025-12-26

**Authors:** Lydia Poole, Ruth A. Hackett

**Affiliations:** 1 Department of Psychological Interventions, School of Psychology, University of Surrey, Guildford, Surrey GU2 7XH UK; 2 Health Psychology Section, Institute of Psychiatry, Psychology and Neuroscience, King’s College London, London, UK

**Keywords:** Diabetes, Weight discrimination, Biomarkers, Glycated haemoglobin, Depressive symptoms

## Abstract

**Background:**

Weight discrimination is associated with negative psychological and physical health. However, in those with diabetes, a high risk-group for weight discrimination, this association is not well-understood. This study examined the extent to which weight discrimination is longitudinally associated with cardiometabolic risk factors in adults with diabetes.

**Methods:**

Participants were 833 people (aged ≥ 50 years) with diabetes at wave 5 (2010/2011) of the English Longitudinal Study of Ageing (ELSA). Perceived weight discrimination was measured at wave 5. Participants provided outcome data at waves 6 (2012/2013) and 8/9 (2016/17, 2018/2019) including psychological (depressive symptoms), anthropometric (body mass index [BMI]), and cardiometabolic biomarkers (glycated haemoglobin [HbA1c], triglycerides, total cholesterol/high density lipoprotein [HDL] ratio, fibrinogen). Linear regressions controlled for baseline age, sex, wealth, BMI, and baseline measures of the outcome of interest.

**Results:**

Perceived weight discrimination was associated with greater depressive symptoms (B = 1.40, *p* < 0.001), higher triglycerides (B = 0.50, *p* = 0.014), poorer total cholesterol/HDL ratio (B = 0.59, *p* = 0.005), and greater fibrinogen (B = 0.25, *p* = 0.025) at wave 6. Effects were attenuated in fully adjusted models and at wave 8/9. Findings were not replicated using a general discrimination measure suggesting results are specific to weight discrimination.

**Conclusion:**

Findings suggest that weight discrimination has deleterious effects on a range of cardiometabolic risk factors in those living with diabetes, at least in the short-term. Interventions targeted to help individuals with diabetes cope with weight stigma are warranted to help reduce the risk of future diabetes-related complications.

**Supplementary Information:**

The online version contains supplementary material available at https://doi.org/10.1007/s12529-025-10421-7.

## Introduction

Diabetes is a chronic metabolic disease affecting insulin secretion and action. In the UK, approximately 4.9 million people live with diabetes, over 90% of whom have type 2 diabetes [1]. Type 2 diabetes is associated with deleterious health outcomes, with complications including retinopathy, kidney disease and cardiovascular disease [2]. Causes of mortality in those with type 2 diabetes are varied [3]. Globally, 26.8% of deaths in persons with diabetes are attributable to vascular complications [4], with UK estimates suggesting approximately 24% of those with diabetes die from vascular diseases (e.g. ischaemic heart disease, stroke) [3]. Research has highlighted the significance of a number of different risk factors in predicting the onset of type 2 diabetes, including psychosocial, behavioural and physiological [5, 6]. Overall, evidence suggests obesity to be the leading risk factor [7]; indeed, approximately 40% of those living with obesity have comorbid diabetes [8]. Given the relationship between type 2 diabetes and obesity, this population represents a vulnerable group at risk of weight discrimination [9].

Tomiyama defines weight stigma to be “the social devaluation and denigration of people perceived to carry excess weight and leads to prejudice, negative stereotyping and discrimination towards these people” [10]. Data from the UK have shown a linear association between body mass index (BMI) and the prevalence of weight discrimination, with over a third of those with extreme obesity (i.e. BMI > 40kg/m^2^) reporting having been discriminated against because of their weight [11]. Similar trends have also been observed using US data [12]. Meanwhile, analyses using harmonised data of adults aged 50 and older have revealed a higher proportion of older adults living in England to report weight discrimination than their US counterparts [13]. Importantly, in those with type 2 diabetes, weight stigma is frequently experienced. For example, in one US study of 1212 adults with type 2 diabetes, 53% reported a history of weight stigma and 26% of participants reported their weight as a barrier to receiving appropriate health care [14]. Perceived weight discrimination is associated with increased depressive symptoms in cross-sectional and prospective analyses [15] independent of BMI. It has also been found to partly explain the association between obesity and poor quality of life, increased depressive symptoms and reduced life satisfaction [16]. When weight stigma is internalised, it can be particularly detrimental to mental health [17]. In those with diabetes, internalised weight stigma has been associated with higher diabetes-related distress [18].

In addition, previous work has shown weight discrimination to be associated with a range of poorer physical health risk factors. For example, in a sample of adults in the US *without* diabetes, weight discrimination has previously been shown to moderate the effect of high waist-to-hip ratio on high glycated haemoglobin (HbA1c) levels, independent of BMI [19]. Moreover weight discrimination in those living with overweight/obesity has been associated with an increased risk of incident diabetes and myocardial infarction in a survey of over 21 thousand adults participating in the US National Survey of Alcohol and Related Conditions (NESARC) study [20]. In a cross-sectional study of community dwelling adults with poorly controlled type 2 diabetes, weight discrimination was associated with a range of poorer diabetes-related health outcomes including higher HbA1c levels and reduced self-care behaviours [21].

However, the extent to which weight discrimination negatively effects disease biomarkers in those living with diabetes has yet to be prospectively investigated. Cardiometabolic biomarkers measure physiological or pathological conditions and are frequently used in healthcare settings to detect and prioritise clinical risk [22]. A range of cardiometabolic biomarkers have been identified to increase the risk of microvascular complications in those with type 2 diabetes, including adipose, metabolic, lipid and haemostatic factors [23–25]. For example, dyslipidaemia (measured via biomarkers including cholesterol and triglycerides) in conjunction with markers of hyperglycaemia (such as HbA1c) are known to be associated with diabetic kidney disease, diabetic retinopathy and diabetic neuropathy [26]. Moreover, research has highlighted the potential utility of total cholesterol/high density lipoprotein in predicting cardiovascular risk of in type 2 diabetes [27, 28]. Furthermore, the known impact of psychological factors on cardiometabolic health [29, 30] and, in particular, the role of depression in predicting poor outcomes in type 2 diabetes [31], highlights the importance of exploring depressive symptoms alongside other cardiometabolic risk factors.

We therefore aimed to study the association between weight discrimination and a range of biomarkers related to cardiometabolic health in a nationally representative sample of older adults living in England, with diabetes. Specifically, we sought to examine the effect of weight discrimination on later weight, HbA1c, triglycerides, fibrinogen, total cholesterol/high-density lipoprotein (HDL) ratio and depressive symptoms. We hypothesised that those participants reporting a history of weight discrimination would have poorer biomarker profiles and greater depressive symptoms 2 and 6–8 years later.

## Method

### Participants

Participants were core members, aged 50 years and older taking part in the English Longitudinal Study of Ageing (ELSA). Full details of the ELSA cohort are available elsewhere [32]. Importantly for this study, at every wave, participants in ELSA are shown a list of illnesses and asked to self-report any doctor-diagnosed illnesses they have developed since the previous ELSA wave. We used this information to determine all cases with a positive report of diabetes/high blood glucose up to and including wave 5 (2002/03 to 2010/11). This was used in conjunction with a wave 4 (2008/09) HbA1c > 6.5% [33] to identify those participants with pre-existing diabetes at baseline. As such, we aimed to capture all instances of both diagnosed and undiagnosed diabetes in our sample.

### Measures

#### Predictor variables: Weight discrimination

Weight discrimination was measured in ELSA at wave 5 (2010/11) alone, with items developed and used in other cohort studies [34]. Participants were asked how frequently they encounter five discriminatory situations: *“In your day‐to‐day life, how often have any of the following things happened to you:* *1) you are treated with less respect or courtesy; 2) you receive poorer service than other people in restaurants and stores; 3) people act as if they think you are not clever; 4) you are threatened or harassed; and 5) you receive poorer service or treatment than other people from doctors or hospitals.”*. Response options were on a 6‐point scale (“almost every day/at least once a week/a few times a month/a few times a year/less than once a year/never”).

Most participants reported never experiencing discrimination, meaning the data were skewed. Therefore, in line with other ELSA studies on weight discrimination [11, 13, 15, 16], we dichotomised responses to indicate whether or not participants reported discrimination in the past year (a few times or more a year vs. less than once a year or never), with the exception of the item on discrimination from doctors or in hospitals which was dichotomised to indicate whether or not participants had ever experienced this type of discrimination (never vs. all other options). This was done as most participants reported never experiencing medical discrimination. An overall discrimination variable was then created from these binary responses across the five situations (0 = no perceived discrimination in the past year, 1 = yes perceived discrimination in any of the 5 settings in the past year). This overall (non-attribution) specific variable is used in sensitivity analyses (see details on the third sensitivity analysis below). A follow-up question asked participants who perceived discrimination for the characteristic(s) they attributed to their experience to. Participants selected from a list of options including weight, age, sex, and race among others. Participants could choose more than one characteristic from the list. Those who attributed any experience of discrimination to their weight are treated as cases of perceived weight discrimination as in other ELSA studies [11, 13, 15, 16]. There is some evidence that using different coding of perceived discrimination items can influence associations between discrimination and health [35]. Therefore, for use in sensitivity analyses, we derived a binary variable to indicate whether or not participants had ever experienced weight discrimination in any of the 5 settings (never vs. all other response options).

#### Outcome Variables: Cardiometabolic Risk Factors

Outcome variables included weight, HbA1c (%), triglycerides (mmol/l) and fibrinogen (g/l). In addition, the ratio between total cholesterol (mmol/l) and high-density lipoprotein (HDL) (mmol/l) was also derived (total cholesterol/HDL) [36]. Biomarkers were measured using blood collected from the forearm during nurse visits conducted at wave 6 (2012/13) and at waves 8/9 (2016/17, 2018/2019). Due to the funding of ELSA, half the sample had a nurse visit at wave 8 and the remainder had a nurse visit at wave 9; for the purposes of our analyses, we have pooled this data. Participants who had a clotting or bleeding disorder and those on anti-coagulant medication did not provide blood samples. Fasting samples, defined as not eating or drinking for 5 h prior to blood draw, were collected where possible and when not otherwise contraindicated (e.g., > 80 years, known diabetes, frail or unwell, ever had a seizure). All blood samples were analysed at the Royal Victoria Infirmary laboratory in Newcastle upon Tyne, UK; for a detailed description of blood analyses see [37]. BMI was calculated using the standard formula (kg/m^2^).

Depressive symptoms were also included as an outcome in models using scores derived from the Centre for Epidemiological Studies Depression 8-item scale (CES-D) [38, 39]. We computed a summary score by adding responses to all eight dichotomous questions (possible range: 0–8). We used the outcome data at wave 8 in our analyses and repeated with wave 9 data in our sensitivity analyses. The Cronbach’s alpha for the CES-D in this study was 0.790.

#### Covariates

All covariates were selected for inclusion in models based on their known relationship with either the exposure or outcome variables. Sociodemographic covariates were measured at wave 5 and included age, sex and wealth. Age was measured in years and sex was categorised as male/female. Socioeconomic position was included in models as quintiles of net financial wealth, which refers to participants’ gross financial wealth with financial debt subtracted. Wealth is the most robust measure of socioeconomic position in ELSA, and has been found to be more strongly associated with the risk of death than any other socioeconomic position measure in this cohort [40].

Baseline measures of the outcome variables were also included in models as covariates including BMI, HbA1c, triglycerides, total cholesterol/HDL ratio, fibrinogen and CES-D scores using methods described previously. Nurse-visit data was not available at wave 5 and therefore wave 4 (i.e. 1-wave prior to baseline) was used for objective BMI and biomarker covariates.

#### Statistical Analysis

Participant characteristics are presented as means (standard deviations) and numbers (percentages) for continuous and categorical variables respectively. Pearson’s correlation and Chi-square analyses were used to assess the association between weight discrimination and covariates. Hierarchical linear regression was used to assess the relationship between perceived weight discrimination at wave 5 and each cardiometabolic risk factor at wave 6 and at wave 8/9. In line with previous studies [11, 13, 15, 16, 41], we used ‘a few times a year or more vs. less’ perceived experience of weight discrimination as the predictor in our primary models. Given variability in the availability of biomarker data, separate Ns are reported for all models. Complete case analysis was performed throughout. We present minimally adjusted models controlling for age and sex and wealth, as well as fully adjusted models. Fully adjusted models included the covariates: age, sex, wealth, BMI, and the baseline variable of the outcome. We presented our findings in minimally and fully adjusted models given controlling for baseline outcome variables accounts for confounding and thus may produce more conservative estimates that reflect change beyond baseline levels rather than absolute associations. Results for all models are presented as unstandardised beta coefficients (B) with 95% confidence intervals (C.I.). All analyses were conducted using SPSS version 29. Two-tailed tests were used throughout, and the significance level was set at *p* < 0.05, though exact significance levels are reported. Due to multiple comparisons, *p*-values adjusted using the Benjamini Hochberg procedure to decrease false discovery rate are also presented.

#### Sensitivity Analyses

We conducted a range of sensitivity analyses to test the robustness of our findings. The first sensitivity analysis repeated the depressive symptoms model, using wave 9 rather than wave 8 data. The second sensitivity analysis repeated the models using the ‘ever vs. never’ cut-off of perceived weight discrimination (instead of the ‘a few times a year or more vs less than once a year/never’ variable used in the main analysis). The third sensitivity analysis used a non-attribution specific discrimination variable across all possible attributions (e.g., age, race, sex, and weight discrimination amongst others) to predict each of the outcomes. The final sensitivity analysis looked at the associations between perceived weight discrimination and the outcomes in those with self-reported diabetes alone (n = 718) (i.e. not taking into account HbA1c values to define our diabetes sample).

## Results

Participants were 833 people (aged ≥ 50 years) with diabetes at wave 5 (2010/2011) who had data on weight discrimination. Compared to those included in our analytic sample versus those excluded, there was no significant difference in responses on perceived weight discrimination, sex, or BMI. However, our included sample were on average wealthier (x^2^ = 14.48, *p* = 0.006) and younger (*t* = 3.217, *p* = 001) than those excluded. Table 1 reports the characteristics of the sample at baseline. On average, participants were 69.5 years of age and approximately half were female (48.7%). The mean BMI (31.35 kg/m^2^, range = 18.00–58.10 kg/m^2^) of the sample, is characteristic of the type 2 diabetes population. In our sample, ever perceiving weight discrimination was reported by 9.2% (n = 77) of individuals, while more frequent reporting of a few times a year or more affected 7.9% (n = 66) of participants. Perceiving experiences of weight discrimination a few times a year or more was significantly associated with younger age (*r* = −0.21, *p* < 0.001), higher BMI (*r* = 0.38, *p* < 0.001) and lower wealth (x^2^ = 28.58, *p* < 0.001); the same pattern of significant associations was observed when using the ‘ever vs. never’ cut-off of weight discrimination.

**Table 1:** Demographic, and cardiometabolic characteristics of the sample (N = 833)

	Diabetes (N = 833)
Characteristic	Mean ± SD or N(%)
Weight discrimination – a few times a year or more (wave 5)	66 (7.9%)
Baseline sociodemographics	
Age (years)	69.51 ± 8.92
Female (%)	406 (48.7%)
Net financial wealth – quintiles 1 (highest) 2 3 4 5 (lowest)	203 (24.4%) 169 (20.3%) 166 (19.9%) 147 (17.6%) 148 (17.8%)
Baseline cardiometabolic variables	
Wave 5 CES-D (N = 825)	1.80 ± 2.05
Wave 4 Body mass index (kg/m^2^)	31.35 ± 6.23
Wave 4 HbA1c (%) (N = 605)	7.11 ± 0.60
Wave 4 Triglycerides (mmol/l) (N = 611)	2.12 ± 1.10
Wave 4 Total cholesterol/HDL ratio (mmol/l) (N = 611)	3.66 ± 0.98
Wave 4 Fibrinogen (g/l) (N = 596)	3.70 ± 0.99

CES-D = Centre for Epidemiological Studies Depression scale; HbA1c = glycated haemoglobin; HDL = high density lipoprotein

Results from hierarchical linear regression models are reported in Table 2 and revealed that weight discrimination (a few times a year or more vs. less) was significantly associated with a range of cardiometabolic risk factors at two-year follow-up. Specifically, after controlling for age, sex and wealth, perceived weight discrimination was significantly positively associated with depressive symptoms (B = 1.40, *p* < 0.001), BMI (B = 6.88, *p* < 0.001), triglycerides (B = 0.50, *p* = 0.014), total cholesterol/HDL ratio (B = 0.59, *p* = 0.005) and fibrinogen (B = 0.25, *p* = 0.025). In fully adjusted models, weight discrimination remained significantly associated with greater triglycerides (B = 0.51, *p* = 0.006) and total cholesterol/HDL ratio (B = 0.53, *p* = 0.002). Including BMI and the baseline outcome variable significantly increased the proportion of variance explained in predicting the outcome across all models (*R*^*2*^ change values were all significant at *p* < 0.001). At 4/6-year follow-up, using wave 8/9 data, results were largely attenuated, although some associations remained between weight discrimination and greater depressive symptoms (B = 1.04, *p* < 0.001) and higher BMI (B = 6.22, *p* < 0.001) in minimally adjusted models. In sensitivity analyses, results were similar using wave 9 CES-D scores as the outcome variable (Step 1: B = 0.72, *p* = 0.012; Step 2: B = −0.05, *p* = 0.873). Following the Benjamini Hochberg adjustment, all associations remained consistent with the original significance values. Results were also upheld using ‘ever vs. never’ as the measure of weight discrimination (see Supplementary Table 1). In further sensitivity analyses using a composite measure of global discrimination across all attributions, findings were non-significant except in minimally adjusted models to predict depressive symptoms (see Supplementary Table 2). Finally, results were largely maintained in models where we restricted our sample to only participants who self-reported diabetes at baseline, with the exception of the model to predict triglycerides in which findings were attenuated (B = 0.41, *p* = 0.068) (see Supplementary Table 3).

**Table 2:** Prospective associations between baseline weight discrimination (a few times a year or more vs. less) and cardiometabolic risk factors

Model	Outcome	N	Age, sex and wealth adjusted B (95% C.I.)	*p*	*R*^*2*^	Fully adjusted B^a^ (95% C.I.)	*p*	*Adjusted p-value*^*b*^	*R*^*2*^ change	Sig* R*^*2*^ change
Wave 6 outcomes										
1	CES-D	709	1.40 (0.90–1.90)	** < 0.001**	0.110	0.22 (−0.23–0.66)	0.341	0.341	0.300	** < 0.001**
2	BMI	638	6.88 (5.21–8.54)	** < 0.001**	0.159	0.44 (−0.35–1.23)	0.276	0.478	0.670	** < 0.001**
3	HbA1c	381	−0.08 (−0.52–0.36)	0.723	0.044	−0.08 (−0.48–0.32)	0.689	0.795	0.269	** < 0.001**
4	Triglycerides	386	0.50 (0.10–0.89)	**0.014**	0.044	0.51 (0.15–0.86)	**0.006**	**0.018**	0.268	** < 0.001**
5	Total cholesterol/HDL ratio	385	0.59 (0.18–1.00)	**0.005**	0.050	0.53 (0.19–0.87)	**0.002**	**0.006**	0.383	** < 0.001**
6	Fibrinogen	368	0.25 (0.03–0.47)	**0.025**	0.026	0.10 (−0.09–0.30)	0.301	0.301	0.282	** < 0.001**
Wave 8/9 outcomes										
1	CES-D	546	1.04 (0.48–1.61)	** < 0.001**	0.085	0.17 (−0.37–0.71)	0.540	0.540	0.198	** < 0.001**
2	BMI	489	6.22 (4.38–8.05)	** < 0.001**	0.159	0.41 (−0.63–1.44)	0.441	0.551	0.600	** < 0.001**
3	HbA1c	267	0.17 (−0.50–0.85)	0.619	0.030	0.06 (−0.55–0.67)	0.846	0.910	0.237	** < 0.001**
4	Triglycerides	271	0.14 (−0.32–0.60)	0.556	0.014	0.26 (−0.16–0.68)	0.224	0.448	0.219	** < 0.001**
5	Total cholesterol/HDL ratio	271	0.50 (−0.22–1.22)	0.175	0.009	0.45 (−0.21–1.11)	0.184	0.368	0.215	** < 0.001**
6	Fibrinogen	243	0.08 (−0.26–0.42)	0.634	0.024	0.00 (−0.30–0.31)	0.978	0.978	0.232	** < 0.001**

^a^ age, sex, wealth, BMI and baseline variable of the outcome

^b^Benjamini Hochberg adjusted *p*-value to account for multiple testing using fully adjusted model data

N.B. BMI and biomarkers use baseline of wave 4 data, age, sex and wealth use baseline of wave 5

BMI = Body Mass Index; C.I. = Confidence Interval; HbA1c = Glycated haemoglobin; HDL = high density lipoprotein

## Discussion

Weight stigma is pervasive within Western cultures [42] and those living with type 2 diabetes represent a high-risk group for weight discrimination experiences given the high comorbidity with obesity [9]. In this study, we explored the associations between weight discrimination and a range of cardiometabolic risk factors using a cohort of middle-aged and older adults with pre-existing diabetes drawn from the population study, ELSA. Our novel findings suggest that in those living with diabetes, perceptions of weight discrimination were significantly associated with a range of risk factors indicative of poorer cardiometabolic health over time. Findings were attenuated at longer-term follow-up, suggesting further research is needed to understand the temporality of such effects.

Our findings suggest a significant association exists between perceived weight discrimination and later depressive symptoms, but effects were non-significant after controlling for BMI and baseline depressive symptoms. Depression is a known cardiovascular risk factor [29, 43] and is associated with weight discrimination in the general population [15, 16] but less is known about these effects in those living with diabetes. Some evidence has shown weight discrimination to be associated with increased diabetes distress [21], a psychological disorder related to the distress experienced with managing diabetes and its complications [44]. Diabetes-related distress occurs in approximately 20% of patients with diabetes and can have deleterious effects on glucose control [45], suggesting that the psychological effects of living with diabetes can have implications for clinical outcomes. Moreover, in addition to weight discrimination, people living with type 2 diabetes are additionally affected by negative societal attitudes directed towards diabetes (so-called ‘diabetes stigma’), adding to the vulnerability of this group [14]. Diabetes stigma has been associated with both depression and diabetes distress [46], though more research is needed to understand its relationship with weight discrimination. Our findings suggest that the association between perceived weight discrimination and depressive symptoms are interrelated and closely linked with body weight/obesity. Indeed, obesity and depression are highly comorbid conditions [47], with perceived weight discrimination previously reported to mediate this association [16]; findings remain to be replicated in those with diabetes.

As has been reported using general population samples [48, 49], we also found that perceived weight discrimination was associated with weight gain over time in those living with diabetes. This association was attenuated after controlling for baseline BMI in our primary analyses but withstood adjustment in sensitivity analyses using the ‘ever vs. never’ cut-off for weight discrimination, thus indicating lack of power may have been an issue in our primary models. Another possibility is that ceiling effects were involved, given the high levels of obesity at baseline in our sample. Further studies are required using large samples to corroborate these effects in the diabetes population. One possible contributing factor to the negative impact of weight discrimination on obesity comes from the literature on self-directed stigma, which posits that for some, the exposure to ‘anti-fat’ attitudes leads to this stigma becoming internalised, with blame being directed towards the self [50]. Feelings of self-blame and shame have been shown to hinder attempts at weight loss in those living with obesity [51] and internalised weight stigma is known to adversely affect eating behaviour [52]. In those living with diabetes, there is some evidence to suggest that weight discrimination, coupled with diabetes-related stigma, places these individuals at even greater risk of internalisation [14].

Our findings also found negative consequences of perceived weight discrimination for cardiometabolic biomarkers, including triglycerides, total cholesterol/HDL ratio and fibrinogen. However, no effects were found for HbA1c and the effect on fibrinogen was attenuated after controlling for baseline values of fibrinogen and BMI. Others have reported cross-sectional associations between weight discrimination and glycaemic control in people with type 2 diabetes [21], suggesting further work is warranted to replicate our null findings in prospective analyses. Some research has identified the possible role of reduced self-efficacy in explaining medication non-adherence to antihypertensive medication in participants experiencing weight discrimination [53]. Given we did not consider the extent to which individuals were under the care of healthcare practitioners for type 2 diabetes nor the extent to which medication regimes were being adhered to, future studies which explore the indirect pathways linking weight discrimination to glycaemic control are warranted.

Our findings add to a growing literature suggesting that weight discrimination is associated with widespread disruption to physiology [54], and may in part explain the negative consequences of obesity on cardiometabolic risk [55]. Furthermore, weight discrimination has been linked to risk of early mortality, more so than other attributions of discrimination [56], lending support for our findings which were specific to weight discrimination rather than global perceptions of discrimination. Obesity is also likely to be key to understanding the specificity of our findings given the known detrimental impact of excess body weight on diverse physiological systems [57]. Other, possible causal mechanisms include the role of stress and activation of the hypothalamic pituitary adrenal axis with its modulatory effects on the neuroendocrine and immune systems. Indeed, some evidence suggests an association between perceived weight discrimination and hair cortisol, a marker of chronic stress [41], supporting this hypothesis. Importantly, we have previously observed that type 2 diabetes is characterised by widespread dysregulation of biological stress systems and increased exposure to life stress [58]. Indeed, more recently, we have shown that such dysregulated stress responses in those with type 2 diabetes are predictive of weight gain 7.5 years later [59]. Overall, these findings suggest that weight discrimination, in the context of diabetes, may serve to compound pre-existing physiological vulnerabilities to the stress-axis.

There have been calls to tackle the pervasive, negative societal attitudes to overweight and obesity [60] and coupled with efforts to halt diabetes discrimination [61], our findings point to the pressing need for action. Positive wellbeing is known to exert protective effects on both HbA1c [30] and diabetes onset [62], highlighting the potential benefit of interventions in this space. Opportunities to intervene with internalised weight stigma offer another avenue for future research, possibly through the use of cognitive behavioural therapy [63] or through therapy to enhance self-compassion [64, 65]. However, it should be noted such interventions are still under development and require further testing in healthcare settings to establish the longevity of any observed effects [66]. Finally, healthcare education and fostering a weight-inclusive approach to working have been proposed as strategies to intervene with weight discrimination experienced by patients within healthcare settings [67]. This is particularly salient given reports of widespread anti-fat bias among medical doctors [68].

### Strengths and Limitations

There are number of strengths and limitations to this study. Data were taken from a large, nationally, representative study of ageing in the English population, where objective measures of lipids and other biomarkers were collected from nurses using standardised protocols. Using this data, we were able to derive a sample of adults using both self-report and HbA1c data, thereby strengthening our certainty in capturing both diagnosed and undiagnosed cases of diabetes. The longitudinal nature of ELSA has enabled us to study changes over time. Despite our longitudinal analyses we cannot rule out the possibility of reverse causality and previous research indicates that people with poorer wellbeing may be more likely to perceived discrimination [69]. As such, future research testing reciprocal prospective associations between weight discrimination and cardiovascular would assist in clarifying this issue. Moreover, weight discrimination was only captured at a single wave, precluding examination of dynamic effects on cardiometabolic health. Overall, weight discrimination was experienced by 9% of our sample. Low exposure prevalence may produce unstable effect estimates and increases the risk of type II error, as such our findings may not generalise to populations experiencing higher burden of weight discrimination. ELSA is a predominately White sample, and given the increased risk of diabetes in ethnic minority populations [70], our findings are not generalisable to all communities. Further, our measure of weight discrimination was not tailored to this form of discrimination as participants could attribute multiple characteristics for their perception of discrimination. While this may have helped avoid priming, other tools designed to specifically measure weight discrimination (e.g., the Fat Phobia Scale [71]) may have led to different results. In addition, not all participants had weight data available; one possibility is that those who declined being weighed were more troubled by their weight, which if true, could represent a sampling bias. We did not consider maladaptive dietary and eating behaviours in our analyses. However, these could represent methods used to coping with weight discrimination leading to a vicious cycle of further weight gain and distress [10]. Our sample focused on adults in mid to late life and as such their experiences may not reflect those of younger adults. Lastly, due to availability of blood data, we aggregated wave 8 and 9 blood data to increase the sample size of our follow-up; even so, attrition led to small sample sizes for some outcomes, affecting the precision of our estimates and generalisability of our findings.

## Conclusion

In conclusion, this study demonstrates the detrimental effect of weight discrimination in those living with type 2 diabetes, and its far-reaching effects on cardiometabolic health. Future efforts should focus on tackling societal attitudes as well as bolstering individuals to cope with the psychological impacts of living with obesity.

## Supplementary Information

Below is the link to the electronic supplementary material.

Supplementary file1 (DOCX 20 KB)

## Data Availability

ELSA data is available via the UK Data Service.
