# Supplementary material for: Understanding the Relationship Between Weight Discrimination and Cardiometabolic Risk Factors in Diabetes: Findings from the English Longitudinal Study of Ageing (ELSA)
Source: Int J Behav Med. 2025 Dec 26;33(4):496–505. doi: 10.1007/s12529-025-10421-7 (PMC13624010; doi:10.1007/s12529-025-10421-7)
Supplement: Supplementary file 1 [file 12529_2025_10421_MOESM1_ESM.docx]

**Supplementary Table 1. Prospective associations between baseline weight discrimination (ever vs never) and cardiometabolic risk factors**

| Model | Outcome | N | Age, sex and wealth adjusted B (95% C.I.) | *p* | *R^2^* | Fully adjusted B ^a^ (95% C.I.) | *p* | *R^2^* change | Sig *R^2^* change |
| --- | --- | --- | --- | --- | --- | --- | --- | --- | --- |
| Wave 6 outcomes | | | | | | | | | |
| 1 | CES-D | 709 | 1.18 (0.71-1.64) | **<0.001** | 0.103 | 0.17 (-0.25-0.58) | 0.434 | 0.307 | **<0.001** |
| 2 | BMI | 638 | 7.08 (5.58-8.58) | **<0.001** | 0.182 | 0.90 (1.18-1.63) | **0.015** | 0.648 | **<0.001** |
| 3 | HbA1c | 381 | -0.03 (-0.45-0.39) | 0.891 | 0.044 | -0.09 (-0.47-0.30) | 0.658 | 0.269 | **<0.001** |
| 4 | Triglycerides | 386 | 0.51 (0.14-0.88) | **0.007** | 0.047 | 0.50 (0.16-0.83) | **0.004** | 0.266 | **<0.001** |
| 5 | Total cholesterol/HDL ratio | 385 | 0.49 (0.11-0.88) | **0.012** | 0.045 | 0.44 (0.12-0.76) | **0.008** | 0.384 | **<0.001** |
| 6 | Fibrinogen | 368 | 0.21 (0.01-0.42) | **0.040** | 0.024 | 0.06 (-0.12-0.25) | 0.500 | 0.283 | **<0.001** |
| Waves 8/9 outcomes | | | | | | | | | |
| 1 | CES-D | 546 | 0.94 (0.43-1.45) | **<0.001** | 0.085 | 0.25 (-0.24-0.74) | 0.318 | 0.199 | **<0.001** |
| 2 | BMI | 489 | 6.28 (4.64-7.91) | **<0.001** | 0.178 | 0.78 (-0.16-1.72) | 0.105 | 0.582 | **<0.001** |
| 3 | HbA1c | 267 | 0.31 (-0.25-0.88) | 0.278 | 0.034 | 0.15 (-0.36-0.66) | 0.558 | 0.235 | **<0.001** |
| 4 | Triglycerides | 271 | 0.12 (-0.27-0.50) | 0.557 | 0.014 | 0.18 (-0.18-0.53) | 0.331 | 0.218 | **<0.001** |
| 5 | Total cholesterol/HDL ratio | 271 | 0.29 (-0.32-0.89) | 0.352 | 0.005 | 0.25 (-0.31-0.81) | 0.383 | 0.216 | **<0.001** |
| 6 | Fibrinogen | 243 | 0.11 (-0.17-0.39) | 0.434 | 0.025 | 0.02 (-0.23-0.28) | 0.870 | 0.231 | **<0.001** |

^a^ age, sex, wealth, BMI and baseline variable of the outcome.

N.B. BMI and biomarkers use baseline of wave 4 data, age, sex and wealth use baseline of wave 5.

BMI= Body Mass Index; C.I.= Confidence Interval; HbA1c = Glycated haemoglobin; HDL = high density lipoprotein.

**Supplementary Table 2. Prospective associations between baseline overall discrimination (a few times a year or more versus less) and cardiometabolic risk factors**

| Model | Outcome | N | Age, sex and wealth adjusted B (95% C.I.) | *p* | *R^2^* | Fully adjusted B ^a^ (95% C.I.) | *p* | *R^2^* change | Sig *R^2^* change |
| --- | --- | --- | --- | --- | --- | --- | --- | --- | --- |
| Wave 6 outcomes | | | | | | | | | |
| 1 | CES-D | 705 | 0.78 (0.51-1.06) | **<0.001** | 0.110 | 0.19 (-0.04-0.42) | 0.103 | 0.303 | **<0.001** |
| 2 | BMI | 633 | 0.67 (-0.28-1.62) | 0.166 | 0.075 | -0.21 (-0.61-0.20) | 0.325 | 0.756 | **<0.001** |
| 3 | HbA1c | 380 | -0.16 (-0.40-0.09) | 0.206 | 0.047 | -0.16 (-0.37-0.05) | 0.132 | 0.265 | **<0.001** |
| 4 | Triglycerides | 385 | 0.11 (-0.11-0.33) | 0.319 | 0.031 | 0.07 (-0.12-0.25) | 0.499 | 0.268 | **<0.001** |
| 5 | Total cholesterol/HDL ratio | 384 | 0.09 (-0.14-0.31) | 0.465 | 0.031 | 0.12 (-0.06-0.30) | 0.186 | 0.391 | **<0.001** |
| 6 | Fibrinogen | 367 | 0.04 (-0.08-0.16) | 0.550 | 0.014 | 0.02 (-0.09-0.12) | 0.754 | 0.293 | **<0.001** |
| Wave 8/9 outcomes | | | | | | | | | |
| 1 | CES-D | 544 | 0.74 (0.43-1.04) | **<0.001** | 0.097 | 0.26 (-0.02-0.54) | 0.069 | 0.189 | **<0.001** |
| 2 | BMI | 486 | 0.81 (-0.21-1.84) | 0.121 | 0.086 | 0.13 (-0.39-0.66) | 0.617 | 0.673 | **<0.001** |
| 3 | HbA1c | 265 | -0.19 (-0.48-0.12) | 0.226 | 0.034 | -0.14 (-0.40-0.12) | 0.299 | 0.235 | **<0.001** |
| 4 | Triglycerides | 269 | -0.15 (-0.35-0.05) | 0.138 | 0.021 | -0.11 (-0.29-0.07) | 0.239 | 0.214 | **<0.001** |
| 5 | Total cholesterol/HDL ratio | 269 | -0.21 (-0.52-0.11) | 0.206 | 0.008 | -0.05 (-0.34-0.23) | 0.716 | 0.211 | **<0.001** |
| 6 | Fibrinogen | 242 | 0.03 (-0.13-0.18) | 0.729 | 0.024 | 0.05 (-0.08-0.18) | 0.456 | 0.236 | **<0.001** |

^a^ age, sex, wealth, BMI and baseline variable of the outcome.

N.B. BMI and biomarkers use baseline of wave 4 data, age, sex and wealth use baseline of wave 5.

BMI= Body Mass Index; C.I.= Confidence Interval; HbA1c = Glycated haemoglobin; HDL = high density lipoprotein.

**Supplementary Table 3. Prospective associations between baseline weight discrimination (a few times a year or more versus less) and cardiometabolic risk factors, in those with self-reported diabetes (n = 718)**

| Model | Outcome | N | Age, sex and wealth adjusted B (95% C.I.) | *p* | *R^2^* | Fully adjusted B ^a^ (95% C.I.) | *p* | *R^2^* change | Sig *R^2^* change |
| --- | --- | --- | --- | --- | --- | --- | --- | --- | --- |
| Wave 6 outcomes | | | | | | | | | |
| 1 | CES-D | 607 | 1.63 (1.08-2.18) | **<0.001** | 0.130 | 0.44 (-0.06-0.94) | 0.085 | 0.287 | **<0.001** |
| 2 | BMI | 550 | 6.91 (5.10-8.72) | **<0.001** | 0.166 | -0.18 (-1.04-0.68) | 0.681 | 0.667 | **<0.001** |
| 3 | HbA1c | 306 | -1.14 (-0.67-0.38) | 0.596 | 0.051 | -0.14 (-0.61-0.34) | 0.576 | 0.278 | **<0.001** |
| 4 | Triglycerides | 312 | 0.41 (-0.03-0.86) | 0.068 | 0.053 | 0.48 (0.07-0.90) | **0.023** | 0.221 | **<0.001** |
| 5 | Total cholesterol/HDL ratio | 311 | 0.55 (0.11-0.98) | **0.015** | 0.068 | 0.49 (0.10-0.88) | **0.014** | 0.304 | **<0.001** |
| 6 | Fibrinogen | 300 | 0.29 (0.05-0.53) | **0.019** | 0.029 | 0.14 (-0.08-0.37) | 0.204 | 0.273 | **<0.001** |
| Wave 8/9 outcomes | | | | | | | | | |
| 1 | CES-D | 464 | 0.95 (0.36-1.54) | **0.002** | 0.092 | 0.09 (-0.50-0.68) | 0.760 | 0.165 | **<0.001** |
| 2 | BMI | 420 | 6.38 (4.41-8.35) | **<0.001** | 0.171 | 0.15 (-0.97-1.27) | 0.789 | 0.592 | **<0.001** |
| 3 | HbA1c | 214 | 0.23 (-0.53-0.98) | 0.550 | 0.037 | -0.00 (-0.69-0.68) | 0.991 | 0.258 | **<0.001** |
| 4 | Triglycerides | 219 | 0.17 (-0.34-0.68) | 0.516 | 0.013 | 0.36 (-0.12-0.83) | 0.138 | 0.240 | **<0.001** |
| 5 | Total cholesterol/HDL ratio | 219 | 0.51 (-0.30-1.32) | 0.217 | 0.008 | 0.50 (-0.27-1.26) | 0.203 | 0.211 | **<0.001** |
| 6 | Fibrinogen | 195 | -1.11 (-0.48-0.26) | 0.554 | 0.038 | -0.22 (-0.56-0.12) | 0.197 | 0.247 | **<0.001** |

^a^ age, sex, wealth, BMI and baseline variable of the outcome.

N.B. BMI and biomarkers use baseline of wave 4 data, age, sex and wealth use baseline of wave 5.

C.I.= Confidence Interval; CRP= C- reactive protein; HbA1c = Glycated haemoglobin; HDL = high density lipoprotein.
